# A Simple Scoring System Predicting the Survival Time of Patients with Bone Metastases after RT

**DOI:** 10.1371/journal.pone.0159506

**Published:** 2016-07-20

**Authors:** Wen-Yi Zhang, Hui-Fang Li, Meng Su, Rui-Fang Lin, Xing-Xing Chen, Ping Zhang, Chang-Lin Zou

**Affiliations:** Department of Radiotherapy and Chemotherapy, The First Affiliated Hospital of Wenzhou Medical University, WenZhou, China; University of North Carolina School of Medicine, UNITED STATES

## Abstract

**Objectives:**

This study aimed to develop a scoring system to predict the survival time of patients with bone metastases after radiation therapy (RT). The scoring system can guide physicians to a better selection of appropriate treatment regimens.

**Materials and Methods:**

The medical records of 125 patients with bone metastases treated with RT between January 2007 and September 2010 were reviewed retrospectively. Fifteen potential prognostic factors were investigated: sex, age, Karnofsky performance score (KPS), type of primary tumor, resection of tumor before bone metastases, interval between primary tumor diagnosis and diagnosis of bone metastases, Carcinoembryonic Antigen(CEA), lung metastases before bone metastases, liver metastases before bone metastases, brain metastases before bone metastases, stage, T, N, M, and degree of cellular differentiation.

**Results:**

In an univariate analysis, 10 factors were significantly associated with survival time after bone metastasis: sex, KPS, breast cancer, esophageal cancer, colorectal cancer, interval between tumor diagnosis and diagnosis of bone metastases, CEA, lung metastases before bone metastases, T-staging, and differentiation. In a multivariate analysis, 7 factors were found to be significant: sex, KPS, esophageal cancer, colorectal cancer, interval between tumor diagnosis and diagnosis of bone metastases, T-staging, and differentiation. The median survival of all patients with bone metastases after RT was 14.1 months. There were significant differences in the median survival of patients with bone metastases after RT of 4.9 months, 10.5 months, and 29.7 months in groups 1, 2, and 3, respectively (*P*<0.001).

**Conclusion:**

According to this scoring system, the survival time of patients after bone metastasis can be estimated.

## Introduction

Bones are one of the most common organs for metastases in advanced cancer, following lung and liver[1]. Approximately 30–70% of patients with cancer have bone metastases in their lifetime[2,3]. Most patients with bone metastases have the following symptoms: bone pain, impaired mobility, hypercalcemia, pathological fractures and spinal cord compression[4].

Of the current methods for the treatment of bone metastases, namely, radiation therapy (RT), chemical therapy, surgery, radionuclide and hormone therapy, radiation therapy is the most effective[5]. RT may also be administered for pain relief. After RT, approximately 75% of patients with bone metastases achieve pain relief and 50% remain pain free[1]. Thus, the goals of therapy are to relieve pain, improve quality of life, and delay disease progression[6]. There are two types of RT regimen. The short-course RT programs, such as 1*8 gray (Gy) or 4*5 Gy, are appropriate for patients facing a shortened life span. The long-course RT programs, such as 10 *3 Gy in 2 weeks or 20*2 Gy in 4 weeks, are suitable for a longer expected life span. Therefore, a scoring system is needed to estimate the survival time of patients with bone metastases who have received RT. Such a system would also guide the selection of the appropriate RT regimens for individual patients.

Palliative chemotherapy is used to treat metastatic cancer, including bone metastases, with the goal of palliating symptoms and improving survival[7]. However, it has never been shown to improve quality of life in 20% to 50% of patients with incurable cancers[7]. A patient with a longer expected life span who has one bone metastasis may be treated with RT; however, RT may not be sufficient to delay cancer progression. He/she may therefore require addition chemical or other therapy. For those with a shortened expected life span, the aim of RT is pain relief. Due to the shortened expected life span, chemical therapy, with its numerous and serious side effects, need not be administered.

However, to date there has been no scoring system developed to predict the survival of patients with bone metastases following RT because of a lack of proper methodological instruments. Thus, the current study provides a scoring system that allows for the appropriate prediction of patient survival time after RT.

## Material and Methods

The medical records of 125 patients with bone metastases treated with RT between January 2007 and September 2010 were reviewed retrospectively. Patients were treated with RT in the First Affiliated Hospital of Wenzhou Medical University. The data were obtained from patient files and from their caregivers. This study was approved by the Ethics Committee of the First Affiliated Hospital of Wenzhou Medical University, Wenzhou, China. Because most of the patients had died when we retrospected those patients’ records, written informed consents were obtained from immediate relatives for publication of this report. The eligibility criteria were as follows: confirmation of the primary lesions by pathology, verification of bone metastases by magnetic resonance imaging (MRI), and radiotherapy for bone metastases.

RT was performed with 6-MV linear accelerators. X-rays were delivered through two parallel opposed fields, such as anterior and posterior fields. Treatment volumes usually encompassed one normal vertebra above and below the lesions of vertebral metastases or expanded above and below the lesions of other bone metastases by 3 cm. The radiation schedule was 10*3 Gy in 2 weeks.

There were 15 potential prognostic factors evaluated with regard to the survival time after bone metastases treated by RT: sex, age(<60 *vs*. ≥60 years; median age, 60 years), Karnofsky performance score(KPS, <80 vs. ≥80; median KPS, 80), type of primary tumor (breast cancer vs. lung cancer vs. esophageal cancer vs. colorectal cancer vs. other tumors), resection of tumor before bone metastases, interval between tumor diagnosis and diagnosis of bone metastases(<3 years vs. ≥3 years), Carcinoembryonic Antigen (CEA, <4.1 ng/mL vs. ≥4.1 ng/mL; median CEA, 4.1 ng/mL), lung metastases before bone metastases, liver metastases before bone metastases, brain metastases before bone metastases, stage, T, N, M, cellular differentiation degree(poor vs. moderate or well). TNM-staging was evaluated according to NCCN when patients were first diagnosed with cancer. T1-2 and T3-4 represented local early cancer and locally advanced cancer, respectively.

A univariate analysis was used to determine which factors were significant (*P*<0.05). The factors found to be significant in the univariate analysis were included in a multivariate analysis. The multivariate analysis was performed using the Cox proportional hazards model and log-rank tests. Kaplan-Meier analysis was performed to detect any significant differences between each group and to describe the survival curve. Statistical software SPSS version 18.0 was used for statistical analysis.

## Results

A total of 125 patients were entered into this study. Patient characteristics are summarized in Table 1. The median interval between tumor diagnosis and diagnosis of bone metastases was 13.1 months (range, 0–133.9 months). At the time of radiation therapy, the median age was 60 years old (range, 39–83 years old), the median KPS was 80 (range, 50–100), and the median CEA was 4.1 ng/mL (range, 0.3–2537 ng/mL). All patients receive chemotherapy before or after bone metastases(or both). The chemotherapy regimens were selected according to pathological type of primary tumor, previous chemotherapy, the patient’s condition and so on.

**Table 1:** Patient characteristic(n = 125).

Characteristic	No. of patients	Percentage(%)
Sex		
female	44	35.2
male	81	64.8
Type of primary tumor		
breast	16	12.8
Lung	55	44.0
esophagus	10	8.0
colorectal	13	10.4
other	31	24.8
Resection of tumor before bone metastases		
Yes	71	43.2
No	54	56.8
interval between tumor diagnosis and bone metastases diagnosis		
≥3years	28	22.4
<3years	97	77.6
Lung metastasis before bone metastases		
Yes	30	24.0
No	95	76.0
Liver metastasis before bone metastases		
Yes	22	17.6
No	103	82.4
Brain metastasis before bone metastases		
Yes	19	15.2
No	106	84.8
Stage		
Ⅰ or Ⅱ	34	27.2
Ⅲ or Ⅳ	91	72.8
T		
1 or 2	55	44.0
3 or 4	70	56.0
N		
negative	52	41.6
positive	73	58.4
M(0 vs 1)		
0	86	68.8
1	39	31.2
Differentiation		
poor	57	45.6
Moderate or well	68	54.4

Abbreviations: CEA = Carcino Embryonie Antige

The median OS time from bone metastases treated with RT was 14.1 months (range, 0.6–106.1 months). In the univariate analysis, 10 factors were significantly associated with survival: sex, KPS, breast cancer, esophageal cancer, colorectal cancer, interval between tumor diagnosis and diagnosis of bone metastases, CEA, lung metastases before bone metastases, T, and differentiation (Table 2). In the multivariate analysis, 7 factors were found to be significant and were considered for the survival score presented in this study. The following factors were entered into survival score: sex, KPS, esophagus cancer, colorectal cancer, interval between tumor diagnosis and diagnosis of bone metastases, T-staging, differentiation (Table 3).

**Table 2:** Univariate analyses.

Potential Prognostic Factor	hazard ratio	95%CI	p
Sex(female vs male)	0.512	0.343–0.763	**0.001**
Age(≥60 vs<60)	0.742	0.511–1.078	0.118
KPS(≥80 vs <80)	0.578	0.376–0.887	**0.012**
Type of primary tumor			
Breast vs other	0.433	0.237–0.791	**0.007**
Lung vs other	0.968	0.666–1.406	0.865
Esophagus vs other	2.482	1.284–4.799	**0.007**
Colorectal vs other	2.310	1.275–4.185	**0.006**
Resection of tumor before bone metastases(Yes vs No)	0.879	0.605–1.276	0.497
interval between tumor diagnosis and bone metastases diagnosis(≥3 vs <3years)	0.508	0.313–0.825	**0.006**
CEA(≥4.1ng/mL vs <4.1ng/mL)	1.315	0.866–1.997	0.199
Lung metastasis before bone metastases (Yes vs No)	1.574	1.008–2.460	**0.046**
Liver metastasis before bone metastases(Yes vs No)	1.277	0.800–2.040	0.305
Brain metastasis before bone metastases (Yes vs No)	1.274	0.738–2.199	0.384
Stage (Ⅰ or Ⅱ vs Ⅲ or Ⅳ)	0.622	0.396–0.975	**0.038**
T(1 or 2 vs 3 or 4)	0.533	0.361–0.782	**0.002**
N(negative vs positive)	0.875	0.599–1.277	0.488
M(0 vs 1)	0.938	0.628–1.401	0.754
Differentiation(moderate or well vs poor)	0.553	0.379–0.808	**0.002**

Abbreviations: KPS = Karnofsky performance score, CEA = Carcino Embryonie Antige

**Table 3:** Multivariate model.

Potential Prognostic Factor	hazard ratio	95%CI	p
Sex(female vs male)	0.649	0.423–0.996	**0.048**
KPS(≥80 vs <80)	0.488	0313–0.762	**0.002**
Breast vs other	0.827	0.408–0.1.674	0.597
Esophagus vs other	2.215	1.083–4.530	**0.029**
Colorectal vs other	2.478	1295–4.741	**0.006**
Lung metastasis before bone metastases (Yes vs No)	1.492	0.919–2.422	0.105
Differentiation(moderate or well vs poor)	0.544	0.360–0.823	**0.004**
T(1 or 2 vs 3 or 4)	0.645	0.423–0.984	**0.042**
Stage (Ⅰ or Ⅱ vs Ⅲ or Ⅳ)	0.648	0.388–1.084	0.099
interval between tumor diagnosis and bone metastases diagnosis(≥3 vs<3year)	0.562	0.343–0.923	**0.023**

Abbreviations: KPS = Karnofsky performance score

**Fig 1:**
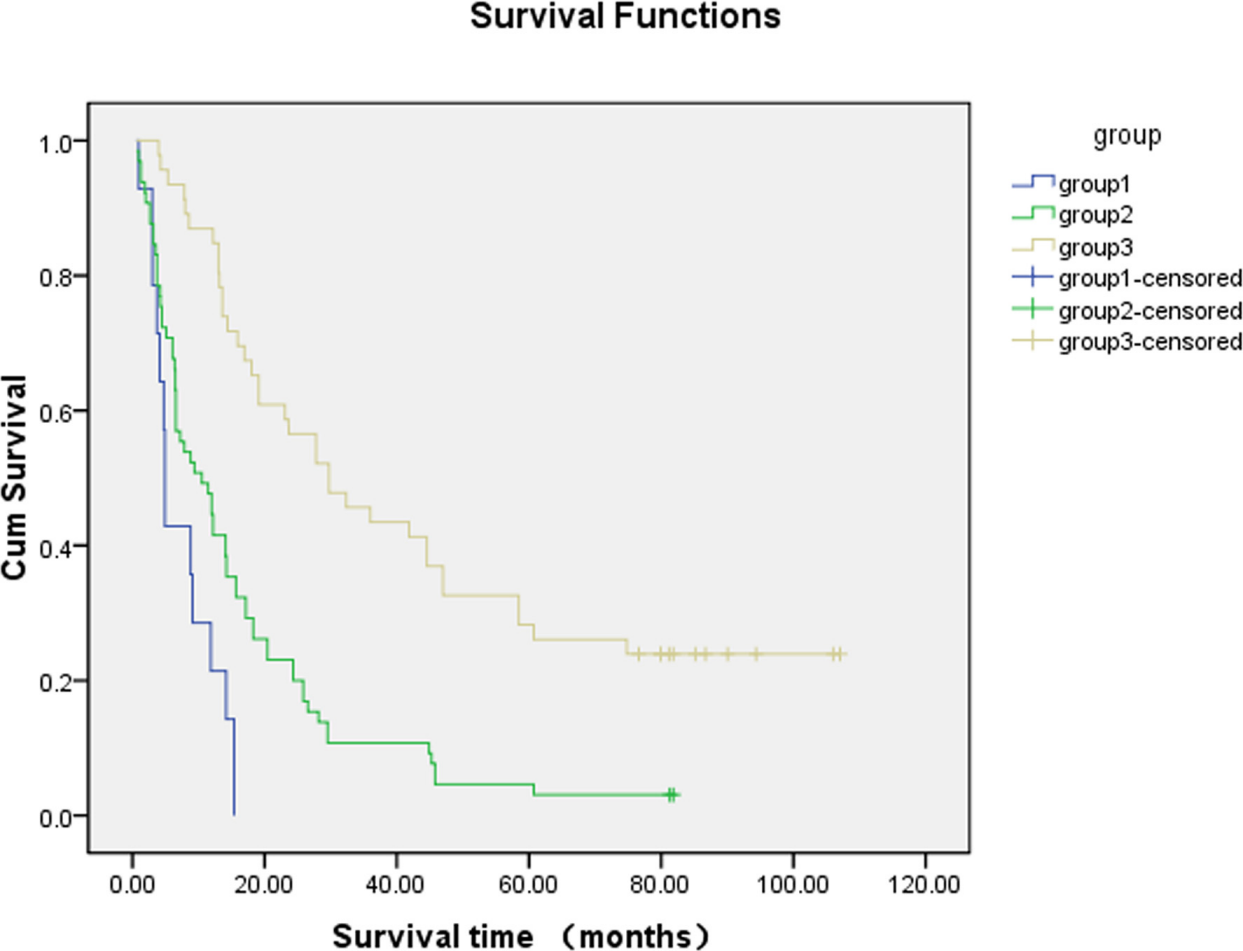
Kaplan-Meier analysis describes survival curve. The median survival of all patients with bone metastases after RT was 14.1 months. The median survival of patients with bone metastases after RT in group 1, 2, 3was 4.9 months, 10.5months, 29.7months, respectively. There was significantly different among those groups. There was significantly different among those groups(*P*<0.001).

With this prognostic model, a survival score was calculated for patients with bone metastases treated by RT. According to 7 significant factors on the multivariate analysis, the score of the factor is 2 or 0 when it’s hazard ratio is<0.5. The score of the factor is -2 or 0 when it’s hazard ratio is >2. The score of the factor is 1 or 0 when it’s hazard ratio is≥0.5 and <1.The score of the factor is -1 or 0 when it’s hazard ratio is >1 and <2. Hazard ratio of sex, differentiation, T-staging, interval between tumor diagnosis and diagnosis of bone metastases is ≥0.5 and <1 according to Table 3. So the patient, who had one kinds of factors including female, High or middle differentiation, T1 or T2-staging, interval between tumor diagnosis and diagnosis of bone metastases ≥3 year, gets 1 point. Hazard ratio of esophageal cancer or colorectal cancer is >2 according to Table 3. The patient, who was diagnosed with esophageal cancer or colorectal cancer, gets -2 points. Hazard ratio of KPS is <0.5 according to Table 3. The patient whose KPS was ≥80, gets 2 points. So the highest aggregate score is 6 points and the lowest aggregate score is -2 points. To verify the validity of the survival score, we divided the patients into 3 groups, the score for group 1 (n = 14) was -2, -1 or 0; the score for group 2 (n = 65) was 1, 2 or 3; the score for group 3 (n = 46) was 4, 5 or 6 (Fig 1, Table 4). The median survival of patients with bone metastases after RT in groups 1, 2, and 3 were 4.9 months, 10.5 months, and 29.7 months, respectively, and there were significant differences among those groups (*P*<0.001).

**Table 4:** Survival scoring and Group.

Potential Prognostic Factor	score
Sex	
female	1
male	0
KPS	
≥80	2
<80	0
Esophagus cancer	
Yes	-2
No	0
Colorectal cancer	
Yes	-2
No	0
Differentiation	
High or middle	1
low	0
T	
1 or 2	1
3 or 4	0
interval between tumor diagnosis and bone metastases diagnosis(every 1 year)	
≥3 year	1
<3year	0
Group	Score range
1	-2,-1,0
2	1, 2, 3
3	4, 5, 6

## Discussion

A large number of patients with bone metastasis survive longer than 6 or 12 months. The long-course RT with 10 to 20 fractions is considered appropriate for patients with an anticipated long lifespan. The short-course RT that is completed in 1 to 5 days is used to treat patients with an expected survival of <6 months and may reduce the cost of therapy[8]. The long RT regimen has been reported to be more effective than the short RT regimen[9]. The short RT regimen gives patients less discomfort than the long RT regimen because of reduced visits to the accelerator and a more rapid analgesic effect. To better select the appropriate regimen, this study developed a scoring system to guide physicians in estimating the survival time of patients with bone metastases. The majority of patients with bone metastases still receive palliative chemotherapy, whose toxicity and side effects reduce the quality of life. Often these patients believe that chemotherapy may save their life, even though they feel stress and anxiety about chemotherapy[10]. The use of chemotherapy in patients with advanced cancer known to have a short life expectancy is associated with an increased risk of requiring cardiopulmonary resuscitation and/or mechanical ventilation, deterioration of the quality of life, discomfort of caregivers/families and higher costs[7,10]. This scoring system is able to categorize patients into three groups to predict survival after treatment with RT, allowing the physician an opportunity to make a better selection of the appropriate regimen.

This scoring system is the first to predict survival time in patients with bone metastases treated by RT. Seven pretreatment variables, including sex, KPS, esophageal cancer, colorectal cancer, interval between tumor diagnosis and diagnosis of bone metastases, T-staging, and differentiation, can be used to forecast survival time in patients after RT to help determine an appropriate radiation schedule and whether palliative chemotherapy should be administered.

KPS has been used to predict survival time in other studies. Tassinari et al[11] developed the palliative prognostic score (PaP score) to predict survival in advanced, pretreated gastrointestinal or non-small cell lung cancer before starting a further line of chemotherapy with a palliative aim. The PaP score discriminated between patients who might benefit from palliative chemotherapy and those who would benefit more from a supportive and palliative approach. The authors also found that KPS was an independent prognostic factor. Fox et al[12] and Li et al[13] reported that a KPS<80 is a hazard to overall survival.

T-staging has been used to predict survival time in other studies. One paper by Nakayama et al[14] evaluating the modified Glasgow prognostic score in patients with advanced head and neck cancer reported that in a multivariate survival analysis, the T (T1, T2, T3, or T4) rather than the N (N0, N1, N2, or N3) classification was significantly associated with disease-free and overall survival.

Sex has been used to predict survival time in other studies. Lee et al[15] reported that the hazard ratio in many types of advanced cancer for male patients is 1.72, with a significant difference in survival between male and female patients.

Methods other than the hazard ratio have been used to predict survival time. Based on a retrospective investigation of 1,852 patients with cancer irradiated for metastatic spinal cord compression (MSCC), Rades et al[9] found the 6 following prognostic factors correlated with improved survival: histology, visceral metastases, other bone metastases, ambulatory status before radiotherapy, interval between tumor diagnosis and MSCC, and time elapsed before the development of motor deficits. However, the authors did not develop a scoring system to predict survival time after radiotherapy of MSCC. In another study, Rades et al[16] developed a scoring system to predict survival time for those patients using percentages rather than a hazard ratio. Chao et al[17] used recursive partitioning analysis (RPA) to predict overall survival of patients with spinal metastases undergoing spine stereotactic body radiation therapy (sSBRT). The RPA analysis used age, time from primary diagnosis (TPD), and KPS to divide those patients into three classes. Although these scoring systems or methods have predicted survival time after spinal metastases, none investigated survival time after bone metastases, including axial and peripheral bone. Furthermore, no study used differentiation to predict survival time.

The patients in group 3 who had a relatively better predicted survival would be expected to receive the long-course RT protocols. Patients in group 1, however, may not benefit from this procedure and may be more appropriately treated with the short-course protocols. Group 2 patients could receive either the long- or the short-course protocol, according to their preference and physical condition.

This study reasonably predicted short- and long-term survival time of patients with bone metastases. However, one limitation of this study is that this scoring system is based on the experience of a single institution, and its validity and usefulness in larger samples needs to be evaluated. This scoring system should also be verified among other institutions. Finally, this scoring system is only appropriate for patients with cancer with TNM-staging.

## Conclusion

The survival time of patients with bone metastases after RT can be predicted by our scoring system, which classifies patients into three groups based on expected survival time. According to the patient's score, the physician can offer a better selection of appropriate treatment regimens such as long- or short-course RT, palliative chemotherapy or not, etc.
